# ‘My health is not a job’: a qualitative exploration of personal health management and imperatives of the ‘new public health’

**DOI:** 10.1186/1471-2458-14-726

**Published:** 2014-07-16

**Authors:** Jennifer CD MacGregor, C Nadine Wathen

**Affiliations:** 1 Faculty of Information & Media Studies, The University of Western Ontario, 1151 Richmond St., North Campus Building, Room 240, London ON N6A 5B7, Canada

**Keywords:** Health behaviour, Health attitudes, Work, Responsibility, Healthism

## Abstract

**Background:**

There is an increasing push in Western healthcare for people to ‘manage’ their health, a key aspect of what has been called the ‘new public health’. It has been argued that this ‘personal health management’ – informal work done to monitor, inform, or influence one’s health – may be a burden, with potential to contribute to poor health outcomes. However, there is little research actually examining perceptions of personal health management and the ‘burden’ of these activities, particularly for generally healthy individuals.

**Methods:**

We conducted exploratory qualitative interviews with 30 generally healthy men and women about their perceptions and experiences of personal health management. Questions focused on health behaviours (e.g., information seeking), as well as feelings about these behaviours and perceptions of the time dedicated to health. Audio-recorded interviews were transcribed and analyzed qualitatively using NVivo 10. Where appropriate, quantitative codes were applied and descriptive statistics are reported alongside qualitative findings.

**Results:**

Participants were generally satisfied with the amount of time spent on their health and few perceived personal health management as a burden. Many participants took issue with the concept of ‘work’ being associated with health and stressed the importance of taking personal responsibility for health.

**Conclusions:**

Our findings suggest that generally healthy people have internalised the notion of the ‘new public health’ and accepted the imperative of personal health responsibility. On the one hand, this bodes well for healthy individuals; their positive attitude may lead to better health outcomes, and the manageable amount of time spent suggests personal health management is unlikely to cause negative health consequences associated with stress. On the other hand, our findings may indicate that other factors, such as social determinants of health, are ignored in health promotion efforts and that those who cannot manage their own health may fall further behind. Future research should continue to explore the time people spend ‘working’ for their health, and how they perceive and respond to ‘new public health’ imperatives.

## Background

Standards for what is considered appropriate and ‘good’ living in Western society have never been higher [1]. Media messages that one can (and should) ‘have it all’ – family, career, money, happiness and health – abound (e.g., [2,3]). Yet, with longer work weeks [4,5], many people struggle to manage the “work-life roller-coaster” [1], ([6] p. 1143). Recently, it has been suggested that health-related activities add to this burden of work, as people juggle things like scheduling and attending appointments, exercising, planning nutritious meals, learning about treatment options, and monitoring the progression of illnesses or symptoms [7,8]. Our research is focused on this issue of ‘personal health management’ , which we define as any informal, unpaid work done to manage, monitor, inform, or influence personal (or family) health outcomes. In particular, we explore how people perceive the time and effort that personal health management takes.

### Time for health

Research has begun to focus on the time people spend on health [9]. For example, recent evidence suggests that American adults spend an average of 42 minutes per week on injury- or illness-related ‘self-care’ such as taking medicine, exercise for medical reasons, etc. [10] and about 2 hours per week participating in sports and fitness [11]. When it comes to accessing health services, Americans spend an average of 121 minutes per visit, including travel, wait time, and actual service, and when time spent by outpatients and their companions is totalled, it amounts to almost an entire work week per year [12]. Not surprisingly, research consistently shows that those in poor health spend the most time on health-related activities [9,10], and women spend more time than men not only for themselves, but for others, especially family [13,14]. Insufficient time is a commonly cited reason for why people do not enact healthier behaviours [15,16] and recent evidence suggests time constraints often mean a trade-off between one healthy behaviour and another (Tumin R, Asti L, Palmisano S, Jena A, Tumin D: Are Food and Fitness Competing Claims on Adults’ Time? Unpublished).

### ‘New’ personal health management

Although nutritious eating and physical activity are the hallmarks of ‘healthy living’ [17], researchers are paying increased attention to three areas of ‘new’ personal health management: 1) health information management (i.e., the storage and organization of health documents or information, either in electronic or paper form) [18-20], 2) health information tracking (i.e., formal/informal, electronic or otherwise monitoring or recording of personal health information, such as symptoms or calorie intake) [21] and 3) health information seeking (conceptualised in a variety of ways, but takes place essentially any time an individual seeks health-related knowledge or information from any source) (e.g., the internet or friends; [22-24]).

Each ‘new’ area has been influenced by information and communication technologies (ICTs), and in particular, by the proliferation of the internet and mobile technologies. New ways to manage health information via (electronic) personal health records proliferate, though widespread use by the public has lagged [25]. Health apps for smartphones are widely available and increasingly popular, allowing tech-savvy individuals to easily track health information and behaviours [21]. Finally, online health information seeking is widespread in North America and Europe [26,27] and the volume of online health information is effectively incalculable.

### New ‘imperatives’ and the ‘new public health’

Although such technologies are generally seen as time-saving advances that make life easier, some have argued that they may actually add to the number of health-related activities that people feel they must undertake and maintain. In their critique of ‘e-health discourse’, Henwood and colleagues identify a ‘connection imperative’ – “a pressure to adopt and use new ICTs to support self care practices” ([28], p. 199). Ziebland ([29], p.1783) notes that “one of the consequences of easier access to health information may be the emergence of a felt imperative to be (or present oneself) as an expert or critical patient, able to question advice and locate effective treatments for oneself”. Similarly, Harris [30] uses the term ‘cyber-burden’ to refer to how the internet itself increases people’s (particularly women’s) burden of work by creating imperatives to be informed regarding one’s (and others’) health.

Such arguments are situated within a broader critical discourse of the ‘new public health’ , the prevailing approach to health promotion in Western societies in which health ‘consumers’ are encouraged to take responsibility for their own health by enacting healthy behaviours and avoiding health risks [31,32]. Often in the name of personal ‘empowerment’ , ‘choice’ , and ‘control’ , this approach shifts responsibility for citizens’ well-being away from governments and healthcare systems and onto individuals themselves [33,34]. Such health promotion strategies instil the desire within individuals to be ‘good’ citizens by self-governing in accordance with health imperatives [34,35]. Thus, in today’s climate of ‘healthism’ [36,37], the “pursuit of healthiness” is paramount ([35], p. 100), and healthy living has become not only a social imperative but a moral one [38,17].

### But is personal health management ‘work’?

There is no doubt that the responsibilities associated with personal health management can, at least according to one definition, involve ‘work’. Planning, shopping for, and preparing healthy meals, going to the gym, obtaining health information online, and caring for sick loved ones are all “work”, i.e., activities “involving mental or physical effort done in order to achieve a purpose or result” [39]. Scholars have argued that informal efforts to care for the health of others (e.g., children, chronically ill elders, etc.) are a form of work that often goes unnoticed and underappreciated [13,40]. Moreover, this ‘unpaid carework’ [30] or ‘health work’ [41] can be associated with considerable caregiver burden and negative health consequences [42,43]. The experience of other, especially the ‘new’ (as above), forms of personal health management – either in addition to, or separate from, these forms of ‘health work’ – is less understood.

Not dedicating enough time to some types of personal health management (e.g., exercise) can be detrimental [44,45]. However, spending too much time may also take its toll. Indeed, long hours spent on paid work are associated with decreases in health and well-being [46,47]. Other findings, however, suggest that how people perceive their work hours is even more important than the number of hours spent [5]. For example, women who are happy with their work hours have better mental health than those who are not [1], and a lack of control over work hours is associated with poorer physical health for men and women if inadequate social support is also experienced [48]. Thus, if personal health management, like other domestic work [6], adds additional burden, it may be that such tasks are also perceived as ‘work’ , defined another way: “a task or tasks to be undertaken; something a person or thing has to do” [39]. Implied in this definition is that personal health management is not approached willingly, rather it is something that people feel they “have to” (i.e., are “obliged or find it necessary to”) “undertake” (i.e., “commit oneself to and begin (an enterprise or responsibility”) [39]. To better understand people’s experiences of personal health management, this exploratory research addresses several related questions: 1) How do people perceive the time they spend on personal health management? 2) What are people’s perceptions of personal health management as ‘work’/‘burden’? and 3) What role do feelings of personal responsibility for health play in understandings of personal health management?

## Method

Because we were interested in people’s subjective perceptions and experiences of personal health management, we used qualitative interviewing so that participants could share their views, in their own words. This manuscript adheres to the RATS guidelines for reporting qualitative studies.

### Interviews

A semi-structured interview guide was designed to explore participants’ health experiences and perceptions of their health-related activities. To address our research questions, specific interview questions focused on the time people spend on their personal health management (in general, and on the ‘new’ personal health management activities defined above), perceptions of burden, and feelings of obligation or pressure to do personal health management. Finally, to further understand participants’ conceptualizations of what they do for their health, they were asked how they felt about two terms, ‘health work’ and ‘personal health management’ , and whether the terms felt appropriate for what they do. The interview guide was piloted on five individuals and revised accordingly to improve question clarity and sequence. Interviews took place in private study or board rooms at five public libraries in two mid-sized cities in Ontario, Canada. All interviews were conducted by the first author in the fall of 2012 and were audio-recorded with permission. Interviews ranged in length from 16 to 81 minutes and averaged 39 minutes (*SD* = 16.92). After the interview, participants completed a short demographic questionnaire and received $25 in appreciation for their time. Ethical approval was received from The University of Western Ontario (protocol #FIMS-2012-13-004R). All participants read an information letter, which included details regarding provisions for anonymity and confidentiality, and provided written informed consent to participate.

### Sample

A purposeful sample of participants was recruited via posters placed in the library branches, in nearby locations (e.g., recreation centre, daycare, retirement home, etc.), and via a local newspaper ad. However, the majority (n = 20, 67%) of participants learned of the study through an ad placed on a local classifieds website. To ensure a varied sample, interested participants were first screened for eligibility via questions regarding their age, marital status, and parental status. Health status was not used as a criterion for eligibility; individuals of any health status were eligible. Sampling continued until theme saturation was reached.

### Analysis

Audio-recordings were transcribed and analyzed qualitatively in several stages using NVivo 10. First, the lead author read all transcripts and created a preliminary coding scheme based on emerging themes and the interview questions [49]. Next, the second author independently examined a subset of transcripts and verified the appropriateness of the coding scheme. The first author then applied the coding scheme by assigning cohesive chunks of text (a phrase or multiple phrases) to themes, revising and adding to the scheme as needed and discussing changes with the second author. To ensure the trustworthiness of the coding, the second author examined and verified all text assigned to the key themes for this paper so that discrepancies could be identified and discussed [50]. Finally, the first and second author discussed the themes and how they were related, and collaboratively chose quotes to accurately reflect the findings. Where appropriate, quantitative (i.e., categorical) codes were applied so that descriptive statistics could be computed and reported alongside qualitative findings.

## Results

### Health status and demographics

In total, 14 men and 16 women participated. Participants ranged in age from 21 to 75 years (*M* = 44.5, *SD* = 16.02). Full demographics are presented in Table 1. Overall, the sample was composed of healthy adults. Most participants mentioned at least one minor health problem or concern over the course of the interview, from very minor (e.g., occasional cold or flu), to non life-threatening conditions (e.g., surgery for leg injury, migraines, etc.), potentially life-threatening conditions (high blood pressure), to (in two instances) conditions that previously threatened the participant’s life (serious gastro-intestinal disease and serious mental illness). One third of participants explicitly and spontaneously identified themselves as having good health.

**Table 1:** Sample characteristics (n = 30)

**Characteristic**	**n (%)**
**Marital status**	
Married/Cohabiting/Common-law	18 (60)
Divorced/Separated	4 (13)
Seriously dating	2 (7)
Single, never married	6 (20)
**Ethnicity**	
White	28 (93)
**Birthplace**	
Canada	22 (73)
**Parental status**	
1 or more child	19 (63)
**Education**	
University/College degree	13 (43)
Postgraduate/Professional degree	3 (10)
Other (e.g., some college, high school only)	14 (47)
**Employment status**	
Currently working at job/business	15 (50)
Other (e.g., retired, homemaker)	15 (50)
**Income**	
< $24,000	7 (23)
$24,000-$39,999	4 (13)
$40,000-$62,999	8 (27)
$63,000-$89,999	4 (13)
$90,000 +	5 (17)
No response	2 (7)

### Perceptions of time spent on health

Participants were asked to discuss the time they spent on health-related activities, and were encouraged to give their perceptions overall (e.g., “a lot”, “a little”, “too much” etc.) rather than specific hour/minute estimates. Responses ranged from “very little” to “a lot”. Nevertheless, most participants perceived the time as an ‘okay’ or ‘good’ amount. Some wanted to spend more, believed they should spend more, or believed one can never spend too much time on health. Others saw health as a “lifestyle” (e.g., P33) they had adopted, rather than something to be fit into their lives. Time spent on health was also seen to vary depending on particular circumstances. For example, if sick, pregnant, or beginning a new health regimen, there was more to do and thus more time was spent:

“*I think it’s okay. … like I don’t feel robbed by it… it depends on the time… when I was dealing with… going through all the testing and stuff that I’ve been going through… like it really put into perspective what people with a chronic illness must go through… how much time they must spend on their health because I was… going to appointments like… you wouldn’t believe right?… definitely, like when there’s a problem… Then you’re like ‘Oh, my health!’ Like ‘I can’t deal with this.’ But on a day to day I would say that I’m pretty comfortable with the amount of time that I spend…*” *(P27)*

When asked specifically about the time that activities such as health information management, tracking, and information seeking take, participants generally saw them as taking up very little or no time. Overall, these tasks took up much less time than tasks related to active living and healthy eating and most participants did not even mention them until specifically prompted. Of the three activities, information-seeking was by far the most common. Participants consulted a variety of information sources, including health care professionals, friends, books etc. Internet use featured prominently in many interviews: 60% (n = 18) of participants were avid users of online health information. Although information seeking could take up a lot of time for some, it was generally seen as something done at one’s leisure when time allowed, rather than an urgent task.

Health information management and tracking were done to a lesser degree, and for some participants, not at all. Most who did record-keeping described the tasks as quick and easy (e.g., simply filing a document after an appointment). Tracking health information was seen similarly, for example, one participant described the ease of entering information into a smartphone app while waiting in line. Another participant who made weekly graphs on the computer to chart his blood pressure described the task as taking “just seconds” (P14).

### Perceptions of ‘burden’ and ‘work’

For most participants (n = 20, 67%), health-related activities were not, and had never been, perceived as a burden. Only two participants (7%) currently considered looking after their health to be a burden and five (17%) described it as a burden in the past. For three participants (10%), whether health was seen as a burden was unclear. There were several explanations provided for why health was not burdensome. For some, it had to do with time. For example, there was no burden because the time spent on health was perceived as manageable, or good health meant that little time had to be spent:

“*Well, generally I am very fortunate at this point and still getting up every morning… none of it is, you know, hard work or a nuisance or… it’s just… commonsensical things that I do, nothing strenuous…*” (P32)

“*I think it’s a reasonable amount of time to spend for the health that I’ve been able to have so I’m happy with that.*” (P30)

“*No because… up until I had [high] blood pressure… I never really had any health issues in my life.*” (P37)

For others, perceptions of health-related activities as enjoyable or important influenced whether health was seen as a burden:

“*And, I think it all matters who it is for how it would feel. Like I remember when [son’s name] was about 18 months and he had a stomach flu really badly… all sorts of bodily fluids all over me and I wasn’t even thinking about that as much as doing whatever I could to get him healthy. Right? That wasn’t a chore or anything, that just, you know, that’s how you do it. There was no thought about, what time this is wasting. There’s only thoughts about, what else can I do?*” (P29)

“*No, because I enjoy it. So if I, probably if I didn’t, for someone that’s trying to lose weight and be healthy, I think to them… it just seems overwhelming. It just seems like a task but to me… I enjoy it, like any time you enjoy doing something you could spend hours doing it and it’s not a big deal.*” (P5)

The questions pertaining to the two terms helped clarify participants’ experiences of burden, and their conceptualizations of personal health responsibility as ‘work’ , in particular. Some participants either had no preference when it came to the terms ‘health work’ and ‘personal health management’ (n = 4, 13%), didn’t like either (n = 2, 7%), or their preference was unclear (n = 5, 17%). Only one individual (3%) preferred the term ‘health work.’ The majority, however, preferred ‘personal health management’ over ‘health work’ (n = 18, 60%). For example, some considered ‘health work’ to be what health professionals do, or found the term not applicable for other reasons:

“*I just consider it to be part of my lifestyle to keep up on basic health rather than it be ‘health work’*.” (P18)

Whereas others were quite impassioned or even seemed to take offense to an association between health and work:

“*…no that’s wrong. It’s not a job to me. My health is not a job. My health is… damn important. You know, it’s… I’m not getting paid to do it, that’s not… it’s a privilege almost, you know you, we have a country where we have that privilege, we can do that. It’s not a ‘health work’ to me, it’s a health… benefit, you know? It’s benefitting me to get healthier. I’m not working, like, ‘health work’? No. That’s, don’t make sense. It doesn’t, the terminology just doesn’t work*.” (P16)

“*No, I don’t like the term if I’m gonna be totally honest. I don’t like the term because you’re making health seem like something like a job. And I don’t think health should be a job. Health is just something that you should take care of. It should be part of what you are, part of what you’re life should be, it should be healthy. You can have a better life if you’re healthy. So I don’t like ‘health work’ because that just means like it’s a job and then nobody wants to do it. And it’s a burden. And health shouldn’t be a burden*.” (P28)

*“I just don’t like the fact that ‘health’ is associated with ‘work.’ If you consider maintaining your health work, you’ve got problems, you know? [laughter] Because… your health is more than just a job… you shouldn’t look at it as a task that you have to do, you know?… It just has a negative psychological impact on me when I see the term ‘work’ associated with ‘health’…”* (P34)

All participants were asked about the term ‘health work’ first and ‘personal health management’ second. And for some, this switch to the second term resulted in a perceptible sense of relief or comfort with corresponding initial reactions such as “*That’s better*” (P16), “*I like that one*” (P3), “*Well, that’s as clear as a bell*” (P8) and “*Yes, and that … seems like it has a nicer feel to it than work.*” (P12). ‘Personal health management’ was not just seen as the better of two poor terms, however. Some participants seemed to really like the term and could articulate why:

“*Yeah, I really like that management word…*” (P4)

“*Yeah, I think that’s a better term. Yeah. And I think it describes more… what it is, right?*” (P27)

*“Yeah, I'm doing ‘personal health management’. That’s [a] beautiful word, the way you put it. It’s excellent… I have to do my health management…”* (P16)

Nevertheless, some pointed out what they didn’t like about ‘personal health management’:

“*That sounds very technical to me [laughter]. That sounds like I need a degree to manage my own health! [laughter]*” (P26)

*“…it’s personal and… that’s a good thing. I like that aspect of it. But it still seems very corporate-sounding… I think we put too much serious emphasis on health and that it’s a ‘go, boom, boom, boom’ kind of thing. Whereas I think if we had a little bit more light-hearted… I think that more people would be ready to do it.*” (P28)

Despite the general preference for ‘personal health management’, a few participants seemed to appreciate the term ‘health work’ because it acknowledged that maintaining one’s health could be difficult:

“*I like it. Because it crosses my mind often. This isn’t easy. Again, it can be more the exercising. It’s not easy, it’s work for me to make it work, you know?*” (P1)

“*Yeah, work. Yeah, ‘health work’. Like, phone the drugstore, order your pills, then you gotta go get them… Yeah, there is work, there’s a workload. I never thought about it.*” (P7)

“*There’s an effort that has to be put into it because, it doesn’t come easily and that we’re so programmed naturally to want convenience and the fast gratification and everything easy, that anything that is worthwhile takes work of some kind.*” (P9)

### Personal health responsibility

Consistent with contemporary discourses of the new public health (e.g., [35]) as well as past research (e.g., [51,52,17]), a belief in personal responsibility for health permeated many of the interviews and seemed to play a significant role in people’s understanding of their personal health management. When asked how they felt about the “health component” of their lives, participants were generally positive, which is perhaps not surprising given their general good health and the explicit interest of some (n = 11, 37%) in health as a topic. Moreover, at some point in the interview, half the participants reported enjoyment from at least one health-related activity. However, for some participants, this question prompted self-evaluation. Thus, the imperative of personal responsibility crept into their responses, even though this was not the intent of the question:

“*I always think I could do more. I always think that you know, if I eat a bag of chips, I always, I don’t feel good about it [laughter]. Because I know it’s not right…”* (P28)

*“I feel pretty good. Could be better. Could be substantially better but I feel really good about it… because how many people at 69 years old can… run six miles?”* (P37)

“*I think I can be pretty positive about it because friends often comment on how healthy we are. So, and, personally I do believe that too, we are having a better lifestyle than the average person [laughs] because… we do not go to Tim Horton’s everyday to stand in the line for that sugary coffee [laughter] or we eat out every week at McDonalds, we just don’t do those things, right?*” (P26)

This imperative of personal health responsibility was also subtly expressed throughout the interviews with words such as “should”, “can’t”, “have to” and “need to”:

“*I know that’s not really the way it’s supposed to work. You should be paying attention to it all the time but…*” (P29)

“*…’cause I’m suppose to know better right? I mean at that time I was like 50 years old and I shouldn’t you know, you shouldn’t let this happen…*” (P30)

“*You have to take a, you have to take an interest in, in your health.*” (P3)

“*…when I haven’t been to the dentist or I haven’t been to the doctor’s… like it wears on me a little bit, right? I’m like ‘Okay, I need to get on that’…*” (P27)

But this imperative was also discussed very explicitly, for example:

“*… as a society, I think there is more pressure coming to bear on people to do so, to just become more aware, because with the increased incidence of diabetes, obesity rising, I’m getting the impression, anyway, that society and the media, there’s a lot of sort of push and promotion towards being proactive about your health, which I think is a good thing.*” (P8)

Unlike the above comment, most participants were explicit about taking personal responsibility without acknowledging a societal ‘push’ or any source of this felt imperative:

“*Yes, it’s on you. Like they say, ‘ignorance is bliss’ or something?… You can’t say, “Oh, I didn’t know that.” You have to look for it…. You’ve got to take a share of, a bit of that responsibility.*” (P11)

“*I, I think it, well… I think it’s the right thing but I also think it’s your responsibility. Like I think everybody should have some responsibility for their own health.*” (P3)

“*Yes. Yes. You know, it’s your body, if you don’t seek it out nobody’s gonna seek it out for you. I feel that you know, if you don’t go to your doctor or even your like, you know, doctor, dentist, massage therapist, chiropractor, whatever you do you’re not going to get, you’re not going to get the healthcare you need if you’re not seeking it out yourself. Nobody’s going to come to you.*” (P20)

Furthermore, when asked if they ever felt obligated or pressured (e.g., by society or the media) to do ‘new’ personal health management activities, many resisted the notion of this influence, and suggested their motivation was primarily personal:

“*No, I think just for my own, my own information … my own benefit… I wouldn’t say that there’s any pressure*.” (P27)

“*I don’t feel obligated, I feel motivated, would be a word I would use*.” (P23)

“*Oh, no… It’s all personal, myself*.” (P4)

“*Umm, no I don’t think so. I think it’s… just mostly for me…*” (P21)

Interestingly, the theme of responsibility sometimes arose within discussion of the health terms, indicating that conceptualizations of work, personal health management, and personal responsibility for health were intertwined:

“*That’s a good one. It is management and you are managing your personal health. So, like we said in the beginning, we have to be the ones that are proactive. I feel, for my health, I’m responsible for my health and being proactive. I can bring in the doctor, I can bring in the naturopath, I can bring in the gym, but I have to be the one to say, “This is what I want for me and I wanna manage my health” instead of looking for someone to help me when it stops being what I want it to be.*” (P9)

“*Because what are they trying to tell us, that being healthy is a lot of work? I mean, in the end, I think it’s more an attitude issue than anything else so… if you care about your health, you’ll act accordingly, right?*” (P26)

## Discussion

Although past research suggests that the burden of health-related activities for chronically ill individuals (and those who care for them) (e.g., [9]) is considerable, the experience of personal health management for the self-described generally healthy person appears to be quite different. Overall, our participants were satisfied with the amount of time they spent on health-related activities and did not perceive them as a burden. The new public health and patient empowerment discourses have been criticised for assuming that people appreciate and desire the ‘empowerment’ of taking control of their own health [53,54]. Yet, our findings actually support the notion that people have accepted and even embrace this responsibility of enacting a variety of traditional healthy behaviours as well as ‘new’ types of personal health management. Consistent with past research, participants consulted a variety of information sources [23,24], especially the internet [26]. There were no clear instances in our data of participants actively questioning or resisting the imperative of personal responsibility for health.

Perhaps our most interesting and novel findings relate to participants’ conceptualizations of health as ‘work’. We gave participants no definition of ‘work’ , and yet they reacted to the term strongly, imbuing it with their own meaning. Although many participants saw their health as ‘work’ in one sense – that is, requiring effort – they very much resisted the notion of ‘work’ in another – that personal health management was labour or toil, or that it could be likened to ‘a job’. Overall, participants much preferred the term ‘personal health management’ because it more accurately reflected their health behaviours, without the negative connotations of ‘health work’; however, it was interesting to note some resistance to this term as being “technical” or “corporate”.

### Rejecting health as ‘work’ and accepting ‘personal responsibility’

There are many possible explanations for why people may reject the association of ‘health’ and ‘work’ and internalise the imperative of personal responsibility for health. It may be that, over time, persuasive health promotion messages have shaped people’s approach to personal health management (for a related argument regarding long work hours see Van Wanrooy and Wilson [5]). For example, Henwood et al. ([51], p. 2026) point out that there is “a steady stream of healthy choice messaging from governments and healthcare providers that encourages us to avoid risky behaviours and engage in healthy practices”. These messages include slogans like ‘*It’s Your Health*’ [55] and ‘*Your Health, Your Choices*’ [56]. Similarly, in her discourse analysis of women’s magazines, Roy ([57], p. 463) finds that “reflecting and reinforcing the discourse of healthism, women’s magazines consistently present health as an important individual responsibility and a moral imperative”. These messages emphasise that good health is a ‘choice’ within the power of all citizens to make, a position that, according to our research, is at odds with the notion of ‘work’ as an unpleasant job people *must* do. An explanation of societal influence is consistent with research showing that subjective norms (i.e., beliefs about whether or not others think one should do something) can be a potent determinant of health-related behaviour [58]. More broadly, it is also consistent with research showing that people are motivated to view the way society ‘is’ , as the way it ‘ought to be’ – to consider the current state of affairs to be the most desirable and reasonable way it *could* be, a motivated tendency driven by the desire to justify one’s socio-political systems [59]. Strong societal pressure may also cause people to ‘buy in’ and conform in order to avoid the stigma of not caring enough about their health or doing all they can to improve it. As one of our participants put it, “…*you don’t want to seem like an idiot when people are talking about this stuff and say ‘oh, I don’t care’. Because, in the end, you realise that you look like a clown, that other people maybe take better care of their health, put more effort into understanding this stuff*” (P13).

In addition to societal pressure, our findings can be explained by the human desire to perceive one’s actions as self-determined. Autonomy (i.e., volition, the “desire to self-organize experience”) ([60], p. 231) is seen as one of three basic psychological needs required for good health and well-being. Not only do people function better and experience better outcomes when free to act autonomously [60], they are also particularly motivated to view their actions as freely chosen and to reassert a sense of autonomy once threatened [61]. For our participants, the term ‘health work’ seemed to threaten perceptions of being free, autonomous individuals. What resulted was rejection of an association between ‘health’ and ‘work’ , and the reaffirmation that one’s personal health management was not only a self-determined choice, but a responsibility taken on – in some cases, happily and even proudly. This interpretation is consistent with the notion of citizens ‘willingly’ conforming and self-regulating themselves in accordance with the new public health [35].

### Implications

Our findings can be viewed in several ways. First, positive attitudes toward health have been shown to independently predict better health outcomes [62], indicating that for this healthy group of people, ownership of their own health and the active steps taken to maintain it should bode well. Second, because some types of personal health management are beneficial (e.g., exercise), the finding that people don’t mind spending time on them may mean the behaviours are more likely to occur, as are the presumed health benefits. The benefits of some types of personal health management, however, especially those we have identified as ‘new’ (information management, tracking, and seeking), are not well-understood; thus, whether their widespread acceptance and adoption should actually be encouraged is unclear. Finally, the finding that people generally don’t feel burdened by personal health management may mean such activities are unlikely to increase the negative health consequences associated with stress [63], at least for generally healthy people.

Our findings make important contributions to existing discourses on the new public health, patient empowerment, and personal health responsibility. As Kay et al. ([59], p. 421) note, the tendency to justify one’s current socio-political systems – exemplified in our finding that participants seemed comfortable with the new public health imperative of personal responsibility – has “profound implications for the maintenance of inequality and societal change”. When people see the way things are as the way they should be, they stop advocating for change [59,64]. In this case, that may mean that established social determinants of health such as unemployment, and income and education inequality, are dismissed, and the notion that some aspects of health are beyond one’s personal control is underrepresented in discussions of health promotion [35]. Instead, strategies construing good health as a matter of ‘choice’ , and poor health as personal ‘failure’ to choose wisely, may be favoured, leaving those who struggle to meet even basic needs like housing inadequately supported (Harris R, Wathen CN, MacGregor JCD, Dennhardt S, Naimi A, Ellis K: ‘Blaming the flowers for wilting’: Idealized aging in a health charity video, submitted).

Unlike much research on health behaviour that focuses on the experiences of ill individuals [22], this work broadens the current understanding of what generally healthy adults do for their health, and, most importantly, how these experiences are perceived by them. In this way, our findings also contribute to understandings of the ‘patient-as-person’ dimension of patient-centred care, which argues that the “psychological world” of individuals and the meaning they ascribe to health and illness are key to providing care ([65], p. 1089), [66]. We argue that perceptions of time, burden, and responsibility are important pieces of people’s overall experiences of illness (and health) and should be part of the ‘story’ that health care providers attempt to understand [67].

Moreover, to our knowledge, this work is the first to qualitatively examine how people perceive the time they spend on their health, and in particular, how a broader range of personal health management activities, including information management, tracking and seeking, are experienced. Our qualitative approach to these issues has advantages over other, more commonly used methodologies, such as time studies. As technologies continue to advance, and health promotion messaging evolves, it is our hope that future research will continue to explore – and perhaps challenge – how people ‘work’ for their health.

### Limitations

We purposefully sampled generally healthy adults, and the generalisability of our findings cannot be extended to other groups. For example, had we sampled only individuals with chronic illness, or their caregivers, our results regarding perceptions of time and burden likely would have been very different. Furthermore, despite research suggesting that women tend to do more personal health management for themselves [26] and their families [68,30,14], we did not find evidence of significant sex differences. It might be that our sample was too varied to detect them. For example, if we had solely interviewed parents of young children, we may have found higher levels of time spent and burden for women [69]. Second, we asked people to make estimates of the passage of time, which are known to be influenced by a variety of factors ([e.g., [70]). Thus, although our intent was not to obtain accurate estimates of time spent on health, we do not know how participants’ experiences of time and burden correspond to actual time spent. Future research to address these issues could include the use of representative samples and diary recording of time spent on personal health management. Finally, with this exploratory study, we have but scratched the surface of people’s perceptions of time, work, and personal responsibility for health. Future, more in-depth analyses of these issues, with attention to both ‘new’ and traditional types of personal health management, are warranted.

## Conclusion

Our findings suggest that generally healthy people have internalised the ideals espoused by the ‘new public health’ and accepted the imperative of personal health responsibility. Unfortunately, this may indicate that other factors, such as social determinants of health and shared/communal approaches to health decision-making [53,71], are ignored by those engaged in health promotion activities, and the pressure to be an ‘empowered’ and ‘healthy’ citizen/patient may mount. Thus, the new public health is reinforced and resistance to this approach to health promotion – if desired – may be increasingly difficult. The assumption that health is (and should be) within all citizens’ personal control could lead to a variety of negative outcomes such as inappropriate government cuts to health services and self-blame or stigmatization for those who cannot meet the high standards of healthism and ‘good’ citizenship [35,71].

## Competing interests

The authors declare that they have no competing interests.

## Authors’ contributions

JCDM and CNW conceptualized and designed the study and conducted data analysis. JCDM conducted the qualitative interviews. JCDM wrote the initial draft and CNW made critical revisions and important intellectual contributions. Both authors read and approved the final manuscript.

## Pre-publication history

The pre-publication history for this paper can be accessed here:

http://www.biomedcentral.com/1471-2458/14/726/prepub
